# Dengue virus type 3 infection in a traveler returning from Costa Rica to Japan in 2023

**DOI:** 10.1186/s41182-024-00620-5

**Published:** 2024-08-01

**Authors:** Tadahiro Sasaki, Ryo Morita, Ikuko Aoyama, Takashi Baba, Tetsushi Goto, Ritsuko Kubota-Koketsu, Yoshihiro Samune, Emi E. Nakayama, Tatsuo Shioda, Michinori Shirano

**Affiliations:** 1 Department of Viral Infections, Research Institute for Microbial Diseases, Osaka University, Osaka, 565-0871 Japan; 2 Department of Infectious Diseases, Osaka City General Hospital, Osaka, 534-0021 Japan; 3 Department of Infectious Diseases, Osaka City Juso Hospital, Osaka, 532-0034 Japan; 4 Virology Section, Division of Microbiology, Osaka Institute of Public Health, Nakamichi 1-3-3, Higashinari-Ku, Osaka, 537-0025 Japan

**Keywords:** Dengue virus type 3, Phylogenetic tree, Costa Rica

## Abstract

The number of dengue cases has increased dramatically in recent years. In Latin America, the number of cases and deaths in 2023 was the highest ever recorded. We report on a patient who had been infected with dengue virus during his stay in Costa Rica in September 2023, and developed the disease after returning to Japan. Plasma obtained from the patient was used for diagnosis and dengue virus serotyping by real-time PCR. The nucleotide sequence of the envelope region of dengue virus was then determined by the direct sequencing method, and this sequence was used for phylogenetic analyses. The patient was found to be infected with dengue virus type 3 genotype III. The sequence from the present case was more homologous with sequences registered in Florida, USA, associated with travel to Cuba in 2022 than with sequences registered in Costa Rica 10 years ago. The Pan American Health Organization reported that only dengue virus type 1 and 2 cases were reported in Costa Rica in 2019–2021, whereas dengue virus type 3 and 4 cases started being reported in 2022. In 2023, the reported numbers of cases with dengue virus types 3 and 4 exceeded those of dengue virus types 1 and 2. In addition, regional differences in endemic strains have been observed in Costa Rica. Our findings suggest that the dengue virus type 3 that infected the patient was more likely an influx of a strain that had been circulating in Caribbean countries such as Cuba in recent years, rather than a re-emergence of an indigenous virus in Costa Rica. The serotypes of dengue virus prevalent in Costa Rica have been changing since 2022. All four serotypes were prevalent in 2023, with a particularly sharp increase in the number of cases of dengue virus types 3 and 4. Future monitoring and surveillance are essential because changes in endemic serotypes can cause antibody-dependent enhancement, which can lead to severe dengue disease presentations.

## Background

Dengue virus (DENV), which causes dengue fever and dengue hemorrhagic fever, has been spreading rapidly in tropical and sub-tropical regions. In 2000, approximately, 500,000 dengue infection cases and 900 deaths were reported worldwide [1]; however, in 2023, over 5 million cases and 5,000 deaths were reported [2]. Latin America was particularly affected in 2023, with 4.57 million cases and 2,363 deaths reported the largest number of cases and deaths recorded to date [3]. DENV is transmitted through mosquito bites and is thought to spread to non-endemic areas via infected travelers or carrier mosquitos that travel with travelers. For example, 160 cases of autochthonous dengue fever were reported in 2014 in Japan, a non-endemic area [4]. Therefore, surveillance and monitoring for DENV in patients and mosquitos is important for predicting DENV epidemics. In Japan, under the Infectious Diseases Control Law, when physicians diagnose dengue fever, they are required to immediately report all cases to their prefecture government through public health centers. The information is used for the National Epidemiological Surveillance of Infectious Diseases by the Ministry of Health, Labor and Welfare, being published in the Infectious Diseases Weekly Report and Infectious Agents Surveillance Report from the National Institute of Infectious Diseases (NIID). Mosquito surveys have also been conducted in several local governments in Japan to raise awareness and vigilance against mosquito-borne infections. In the present study, we describe a traveler who was presumed to have been infected with DENV type 3 in Costa Rica in September 2023, having developed dengue fever in Japan upon his return.

## Main text

**Fig. 1:**
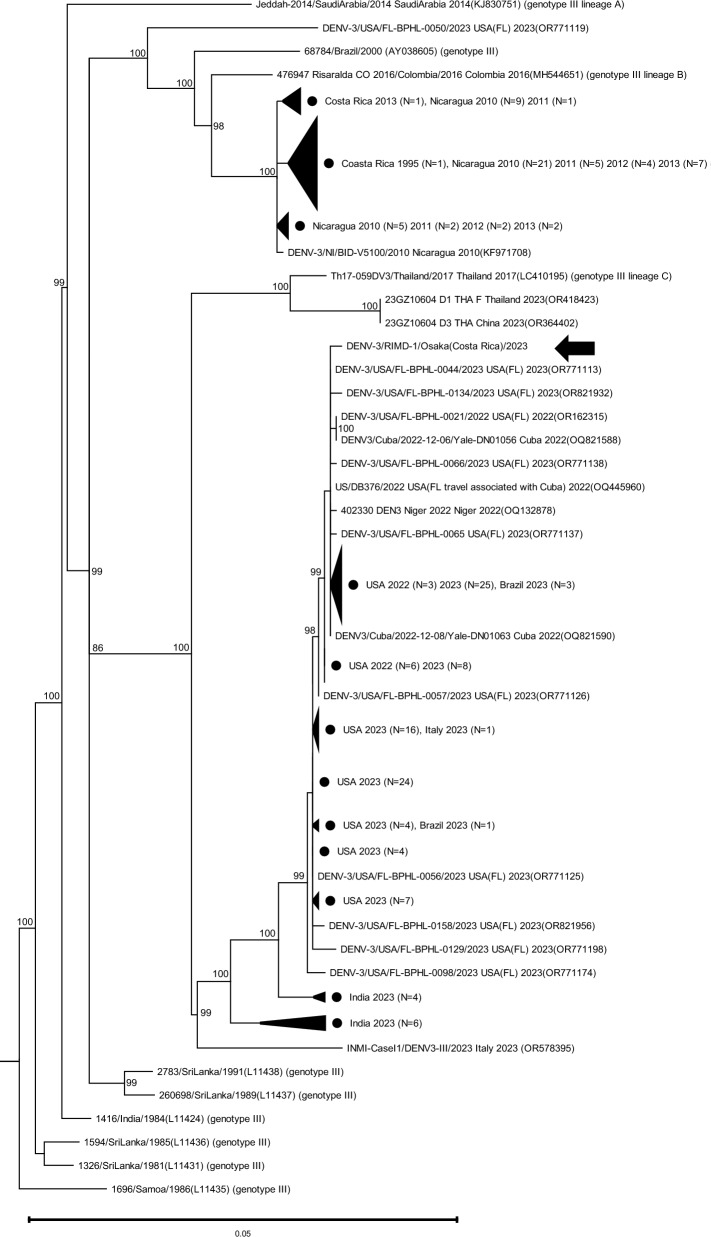
A phylogenetic tree was constructed using maximum likelihood estimation with a TIM2+ F + G4 model, with 1000 bootstrap replications (< 80% values omitted). Since the analysis revealed that the sequence from the present study belonged to Genotype III, Genotype III was separated and is represented in this figure. Black arrow indicates the sequence identified in the present study. Black circles indicate the compressed sequences

A 57-year-old man visited Ostional, Guanacaste Province, Costa Rica in September 2023 via New York City (USA), San Jose (Costa Rica), and Santa Cruz (Costa Rica). After staying there for 5 days, he returned to Japan via San Jose and New York City. After returning to Japan, he developed a fever and presented to Osaka City Juso Hospital the next day. He complained of fever with chills, fatigue, headache, and skin rash. Blood test results showed a low white blood cell count (2120/μL), slightly low platelet count (197,000/μL), and no elevation in the levels of hepatic enzymes or lactate dehydrogenase. He was suspected to have non-severe dengue fever and was diagnosed with dengue fever on the basis of a DENV Rapid Diagnosis Test Kit (SD BIOLINE Dengue Duo NS1 Ag+Ab Combo (Abbott); NS1 positive and IgM/IgG negative). He was given a saline drip and acetaminophen. He claimed to have been bitten by several mosquitoes during his stay in Ostional. Two days after fever onset, blood specimens were collected and sent to the Osaka Institute of Public Health, where real-time polymerase chain reaction (PCR) was performed according to the manual of NIID [5–8] for diagnostic confirmation and determination of the viral load and serotype. The results of real-time PCR indicated that the patient’s plasma was positive for DENV-3 (viral load: 7.8 × 10^9^ copies/μL) and negative for Chikungunya virus, Zika virus, and West Nile virus. Specimens were also sent to Osaka University, where the nucleotide sequence of the envelope region was determined by direct sequencing using specific primers [9]. A phylogenetic tree was generated from the obtained sequence and related sequences retrieved from GeneBank using IQ-TREE [10]. The phylogenetic tree was constructed using the determined sequence and sequences of the envelope region of DENV-3 isolated from humans registered in GenBank according to the following criteria: (1) sequences sampled in 2023 (158 sequences), (2) top 30 sequences with high levels of similarity to the patient’s viral sequences based on BLAST analysis (14 sequences were excluded due to being duplicates from the other criteria), (3) sequences registered as Costa Rica-derived strains (two sequences), (4) sequences sampled since 2010 in Nicaragua, which is adjacent to Costa Rica (59 sequences), and (5) sequences listed in the literature [11, 12] as reference sequences (28 sequences). The tree showed that the present strain was classified as a genotype III lineage C virus, which was prevalent in Asia in the 2010s [12] and was closely related to the OQ445960 sequence isolated in Florida, USA (travel associated with Cuba) in 2022 and the OR771113 sequence isolated in Florida, USA in 2023, with 99.86% nucleotide identity (Fig. 1). The sequences isolated from Costa Rica and Nicaragua used in our analyses were reported over 10 years ago, and no sequences have been registered since 2014. In addition, no sequences of isolates from Panama, another neighboring country, were registered. The sequences reported from Costa Rica and Nicaragua were also of genotype III; however, those sequences were of Lineage B, which was endemic in Latin America in the 2010s, and they showed low levels of homology to sequences of recently distributed strains, suggesting that the present virus was more likely an imported virus from Caribbean countries rather than a re-emerging virus from Costa Rica or its surrounding countries. Of course, since no sequences of DENV-3 strains isolated from Costa Rica in recent years have been registered, this study cannot determine that this patient was infected in Costa Rica. However, considering the patient’s length of stay in Costa Rica and awareness of mosquito bites, we suggest the possibility of infection in Costa Rica rather than during transit in the USA. The Pan American Health Organization reported 30,649 cases of dengue virus in Costa Rica in 2023, with 2712 confirmed cases (629, 429, 687, and 961 with DENV-1, DENV-2, DENV-3, and DENV-4, respectively). Only DENV-1 and DENV-2 were reported in 2019 (131, 233, 0, 0), 2020 (272, 153, 0, 0), and 2021 (215, 37, 0, 0). All serotypes were reported in 2022; however, DENV-1 and 2 remained predominant (319, 216, 1, 15) [13]. This suggests that DENV-3 and 4 spread rapidly in Costa Rica after 2022. The distribution of serotypes differed by region in Costa Rica: DENV-3 was highly prevalent in the provinces of Heredia (51.8%) and Guanacaste (40.4%), which the present patient had visited. DENV-3 was also prevalent in San Jose (34.9%) and Alajuela (33.1%), which are in the northwestern part of Costa Rica. Meanwhile, its prevalence was low in Limon (1.2%) and Cartago (9.4%), which are located in the eastern area [5]. In contrast, DENV-4 was prevalent in Limon (73.6%), Puntarenas (50.1%), and Guanacaste (43.7%) [5]. The lowest prevalence of DENV-4 (10.0%) was reported in Heredia, but this proportion was still approximately nine times higher than the lowest prevalence of DENV-3 in Limon (1.1%) [5]. This suggests that DENV-3 has not yet spread to certain areas of Costa Rica, whereas DENV-4 has already spread to all areas. In addition, the regional differences in the prevalence of each serotype suggest that DENV-3 and DENV-4 entered Costa Rica at approximately the same time around 2022, but with different routes of entry and subsequent expansion.

## Conclusion

The patient was infected with dengue virus type 3 genotype III Lineage B. The sequence of the envelope region in the present case showed higher homology with sequences registered in Florida, USA associated with travel to Cuba in 2022 than with sequences registered in Costa Rica 10 years ago. The resumption of human travel after COVID-19 may have led to an influx of DENV-3 and 4 in Costa Rica. It is known that secondary infection with a different serotype of DENV can induce antibody-dependent enhancement and cause severe disease such as dengue hemorrhagic fever. The current situation in Costa Rica suggests the possibility of a shift in endemic strains from DENV-1 and 2 to DENV-3 and 4, which highlights the importance of further surveillance and epidemiological studies on dengue infection in the future.

## Data Availability

Upon reasonable request, data and materials are available from the corresponding author.
